# Clinical and Genetic Diversity of *PMP22* Mutations in a Large Cohort of Chinese Patients With Charcot-Marie-Tooth Disease

**DOI:** 10.3389/fneur.2020.00630

**Published:** 2020-07-03

**Authors:** Xiaoxuan Liu, Xiaohui Duan, Yingshuang Zhang, Dongsheng Fan

**Affiliations:** ^1^Department of Neurology, Peking University Third Hospital, Beijing, China; ^2^Department of Neurology, China-Japan Friendship Hospital, Beijing, China; ^3^Key Laboratory for Neuroscience, Ministry of Education/National Health Commission, Peking University, Beijing, China

**Keywords:** CMT, PMP22, HNPP, DSS, molecular diagnosis, phenotype

## Abstract

Charcot-Marie-Tooth (CMT) disease is a clinically and genetically heterogeneous group of inherited neuropathies. The purpose of this study is to identify the clinical and genetic diversity of peripheral myelin protein 22 (*PMP22*) in Chinese patients with CMT disease and evaluate their correlations with the clinical manifestations. Using the multiplex ligation-dependent probe amplification (MLPA) technique and Sanger sequencing of *PMP22* in a cohort of 465 Chinese families between 2007 and 2019, we identified 137 pedigrees with *PMP22* duplications (29.5%), 26 pedigrees with *PMP22* deletions (5.6%), and 10 pedigrees with point mutations (2.2%). By comparing our data with the results from other CMT centers in China, we estimate that the frequency of *PMP22* mutation in mainland China is ~23.3% (261/1120). We confirmed *de novo* mutations in 40% (4/10) of *PMP22* point mutations. We have also identified two severely affected patients who are compound heterozygotes for recessive *PMP22* mutations (novel mutation c.320-1 G>A and R157W mutation) and a 1.5 Mb deletion in 17p11.2-p12, suggesting that c.320-1 G>A might be another recessive allele contributing to DSS in addition to the T118M and R157W mutations. A *de novo* mutation of S79P in *PMP22* was also identified concomitantly with the R94W mutation in mitofusin2 (*MFN2*). Our study highlights the phenotypic variability associated with *PMP22* mutations in mainland China. The results provide valuable insights into the current strategy of genetic testing for CMT disease. NGS technology has increased the potential for efficient detection of variants of unknown significance (VUS) and concurrent causative genes. Greater cooperation between neurologists and molecular biologists is needed in future investigations.

## Introduction

Charcot-Marie-Tooth (CMT) disease is the most common hereditary peripheral neuropathy and is characterized by progressive distal muscle weakness and atrophy, distal sensory loss and diminished deep tendon reflex (1, 2). The most common cause of CMT disease is a large 1.5 Mb duplication of the 17p11.2 region that contains the gene for peripheral myelin protein 22 (*PMP22*), accounting for 70% of CMT1 and 50% of all CMT cases (2, 3). The deletion of this region and point mutations and small deletions in the *PMP22* gene are less common and may cause hereditary neuropathy with liability to pressure palsies (HNPP), Dejerine-Sottas neuropathies (DSS) and CMT1E (4, 5). Indeed, many authors have doubted the existence of DSS as a distinct entity, and DSS has more generally been used to describe a particular demyelinating phenotype of CMT1 with an early disease onset and severe clinical symptoms. Moreover, most *PMP22* missense mutations are transmitted in an autosomal dominant pattern, except for R157W and T118M (6, 7), which have been detected in the homozygous state, indicating the genetic and clinical diversity of *PMP22*-related neuropathy.

Although the structure and function of the PMP22 protein have been investigated for many years, some problems remain to be elucidated. (1) The genetic prevalence difference between European, USA countries and Asian countries, such as Japan and Korea, the frequency of PMP22 duplications is relatively low (8–11). (2) Patients with a 17p11.2 duplication and micro mutations in *PMP22* present with varying levels of severity of the disease. (3) A high proportion of patients with CMT1A present with hearing problems. Therefore, more detailed and reliable data based on a large cohort of patients are needed. Here, we summarize the results obtained from a large cohort of Chinese patients and compare our results with data from other major CMT centers in the northern and southern parts of China. We aimed to investigate the clinical and electrophysiological diversity of variations, including the specific genotypes/phenotypes, in mainland China and hence provide new insights into the phenotypic spectrum of specific genetic subgroups.

## Materials and Methods

### Protocol Approval, Registration, and Patient Consent

The institutional ethics committee of Peking University Third Hospital (PUTH) approved this study (IRB 00006761). Informed consent was obtained from all subjects enrolled in this study.

### Patients

We enrolled 580 patients from 465 unrelated Chinese families with suspected CMT disease or a related peripheral neuropathy between January 2007 and December 2019. We collected data on the clinical features, family history, CMT neuropathy score (CMTNS) and electrophysiological evaluations. For patients with hearing loss, we performed the audiogram, otoacoustic emission (OAE), and auditory brainstem response (ABR). Patients were suspected of having CMT disease if they had a sensorimotor peripheral neuropathy with a positive family history. Patients without a family history were also enrolled if their medical history, neurological examination and results of nerve conduction studies were typical for an inherited neuropathy. Patients with an acquired neuropathy, including inflammatory, immune-mediated, toxic, and metabolic neuropathies, were excluded (12). The diagnosis was based on the criteria recommended by the European CMT Collaborative Research Group. All affected patients were evaluated by at least one participating neurologist.

### Genetic Testing

**Figure 1:**
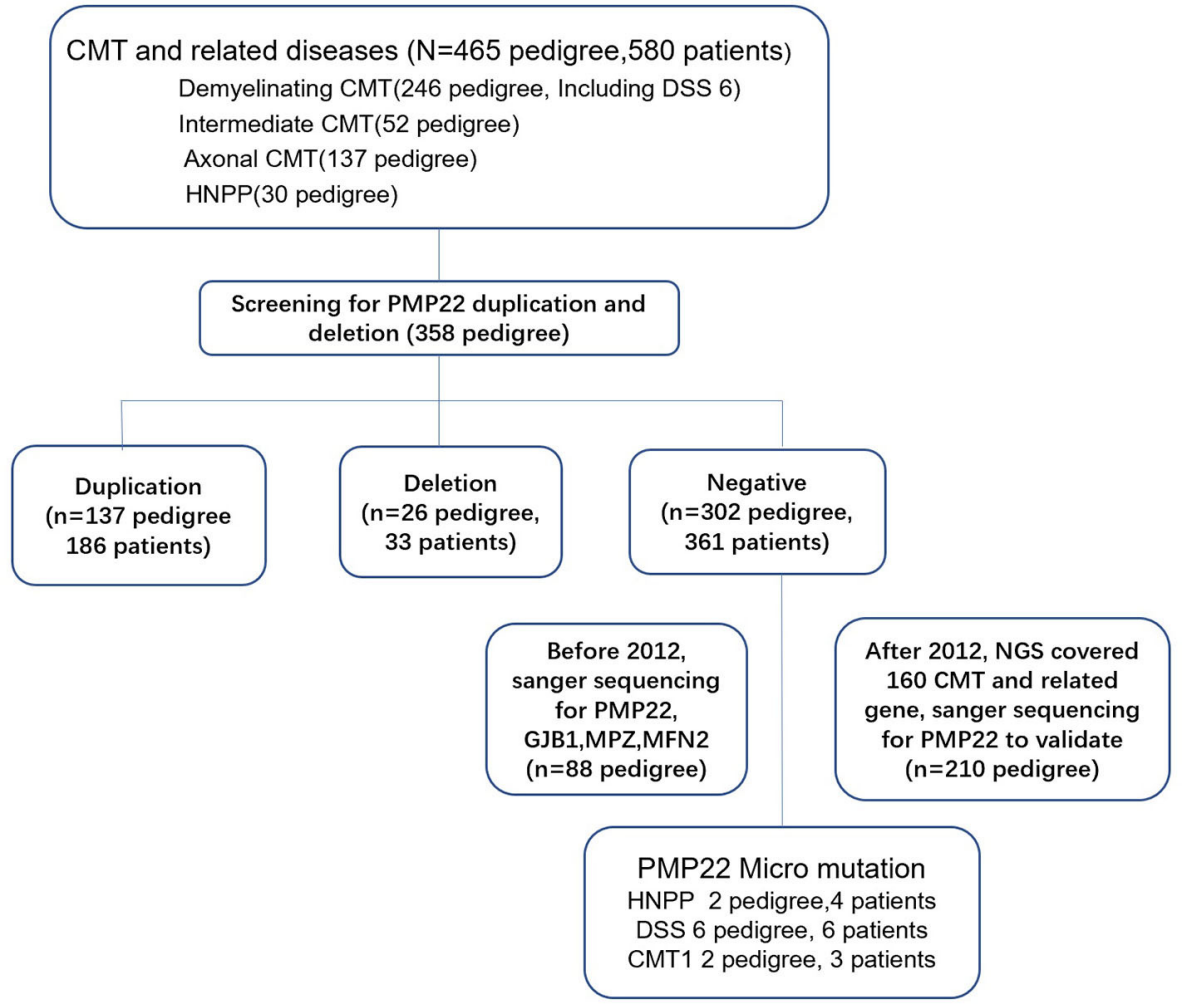
Genetic testing flow chart for patients with CMT disease and HNPP.

Genomic DNA was isolated from peripheral blood samples obtained from the patients using a standard salting method. First, we examined the CMT1A duplications or deletions of 17p11.2-p12 by performing the multiplex ligation probe amplification (MLPA) technique in patients with demyelinating, intermediate CMT disease, part of axonal CMT disease (absent compound muscle action potential (CMAP) or a CMAP <0.5 mV at median and ulnar nerve) and HNPP (358 index patients, see Figure 1). Second, from 2007 to 2012, the regions flanking the coding and splicing sites of the *PMP22* gene were PCR-amplified using intronic primers and directly sequenced using Applera BigDye version 3.1 (Applied Biosystems) and the automated sequencers ABI 3730XL and ABI 3100 (Applied Biosystems) (primers and conditions are available upon request). We also excluded the mutations in myelin protein zero (*MPZ*), gap junction protein beta-1 (*GJB1*), mitofusin2 (*MFN2*) and ganglioside-induced differentiation-associated protein 1 (*GDAP1*) by performing direct Sanger sequencing. After 2012, next-generation sequencing (NGS) covering 160 genes related to CMT disease and related neuropathies was performed for patients with an inherited neuropathy after excluding the presence of a *PMP22* duplication and deletion (see Figure 1). The results of NGS were validated by performing Sanger sequencing.

The assessment of the potential pathogenicity of the CMT mutations was performed using the standard method, including phenotype characterization, absence from the dbSNP (http://www.ncbi.nlm.nih.gov/projects/SNP), 1,000 genomes (http://www.1000genomes.org/), EVUS (http://evs.gs.washington.edu/EVS/) and GnomAD (http://gnomad.broadinstitute.org/) databases, exclusion of Chinese controls, co-segregation with the phenotype in the available familial cases and the *in silico* pathogenicity prediction tools SIFT (http://sift.jcvi.org/www/SIFT_enst_submit.html), PolyPhen (http://genetics.bwh.harvard.edu/pph2/index. shtml) and Mutation Taster (http://www.mutationtaster.org/). The splice site mutations were predicted using the software HSF 3.0 (http://www.umd.be/HSF3/HSF.shtml). Variant classification was based on the ACMG standards (2015) (13), and the pathogenic or likely pathogenic variants were identified accordingly.

## Results

The male to female ratio was 1.3, and the median age at onset was 26.7 years (ranging from birth to 60 years). Based on the electrophysiological criteria, 246 families were diagnosed with CMT1, 137 families were diagnosed with CMT2, 52 families were diagnosed with intermediate CMT disease and 30 families were diagnosed with HNPP. We identified 137 index patients with *PMP22* duplications, accounting for 29.5% (137/465) of the CMT cases and 55.7% (137/246) of the demyelinating CMT cases. In the remaining 109 patients with CMT1, the disease-causing mutation was identified in 22 with the following distribution: 6 mutations in *SH3TC2*, 5 mutations in *GJB1*, 4 in *MPZ*, 2 in *PRX*, 2 point mutations in *PMP2*2, 1 in *GDAP1*, 1 in *HK* and 1 in *NEFL. Twenty-six* index patients with *PMP22* deletions showed manifestations compatible with HNPP, and 10 index patients with *PMP22* missense mutations and small deletions showed phenotypes ranging from CMT1E (2 index patients) to HNPP (2 index patients) and DSS (6 index patients). Thirty percent of the patients with demyelinating CMT disease had no family history. We confirmed *de novo* mutations in 40% (4/10) of *PMP22* point mutations.

### *PMP22* Duplications (CMT1A)

The clinical manifestations of CMT1A are summarized in Table 1. The mean onset age was 24.7 years and ranged from birth to 60 years. Sixty-one index patients (61/137, 44.5%) presented symptoms during the first 2 decades of life. Most patients showed typical phenotypes, including symmetrical weakness and atrophy of the distal part of the limbs. The mean CMTNS score was 14.9. Twelve patients (12/137, 8.8%) displayed hearing problems. They all showed sensorineural hearing loss in the OAE and a prolonged or absent I wave in ABR testing. The average MCV in the median or ulnar nerves was 18.6–19.7 m/s. SCV was absent in 84.7% of the patients. The positive family history, uniformly decreased MCV in the median and ulnar nerves and high possibility of the absence of SCV and MCV in the lower limbs all helped distinguish these patients from the other patients with CMT1.

**Table 1:** Clinical features and electrophysiological results of the index patients with *PMP22* mutations.

	**PMP22 duplications (137)**	**PMP22deletions (14)**	**PMP22 point mutations (10)**
Age of onset, y			
mean (SD) ≤20 y (SD, %) >20 y (SD, %)	24.7 (17.1) 9.5 (5.8, 44.7%) 41.2 (8.7, 55.3%)	24.6 (10.9)	4.7 (3.1)
Family history, *n* (%)	96 (70.1%)	18 (69.2%)	4 (40%)
Typical phenotype, *n* (%)	112 (81.8%)	21 (80.8%)	HNPP 2 (20%) CMT1 2 (20%) DSS 6 (60%)
Hearing problems, n (%)	12 (8.8%)	0	2 (20%)
CMTNS (0-36)Mean (SD)	14.9 (5.9)	8.1 (2.7)	19 (11.4)
MCV (m/s) Median nerve, mean (SD, range) Ulnar nerve, mean (SD, range)	18.6 (7.7, 0–30.4) 19.7 (7.8, 0–36.9)	33.4 (13.6, 21.3–50) 31.2 (5.6, 27–43.2)	Varied
SCV Median nerve, mean (SD, range) Ulnar nerve, mean (SD, range) SCV absent, *n* (%)	22.5 (8.6, 15.5–33.9) 22.4 (8.0, 13.3–31.1) 116 patients (84.7%)	32.8 (18.9, 16.2–55.8) 29.7 (18.5, 18.9–46.9) 0	Varied

**Figure 2:**
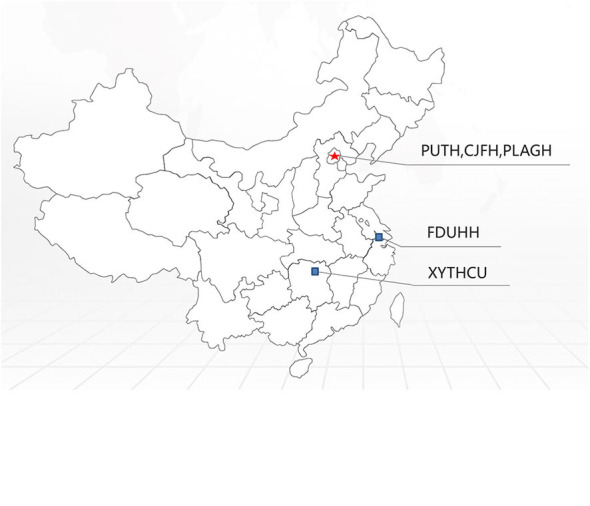
Distribution of *PMP22* mutation frequencies observed in a series of Chinese CMT centers on a Chinese map.

By comparing the frequency of *PMP22* duplications in our data with the frequencies observed in other CMT centers (Table 2 and Figure 2), we estimated that the frequency of *PMP22* duplications in mainland China is ~23.3% (261/1120). We also observed a significant difference between the southern (42/196, 21.4%) and northern parts (95/269, 35.3%) of China. The data obtained from the Third Xiangya Hospital and Huashan Hospital, which are located in the southern part of China, indicated a detection rate of *PMP22* duplications of only 13.5–14%, suggesting that other reasons in addition to ethnic differences should be considered.

**Table 2:** Comparison of *PMP22* mutation frequencies observed in a series of Chinese CMT centers.

**PMP22**	**PUTH (*n* = 465)**	**CJFH (*n* = 126)**	**XYTHCU (*n* = 299)**	**PLAGH (*n* = 82) (11)**	**FDUHH (*n* = 148) (10)**
Duplication, *n* (%)	137 (29.5%)	52 (41.3%)	42 (14%)	10 (12%)	20 (13.5%)
Deletions, *n* (%)	26 (5.6%)	3 (2.4%)	5 (1.7%)	2 (2.4%)	17 (11.5%)
Point mutations, *n* (%)	10 (2.2%)	3 (2.4%)	3 (1%)	0	1 (0.7%)

*PUTH, Peking University Third Hospital; CJFH, China-Japan Friendship Hospital; XYTHCSU, Xiangya Third Hospital of Central South University; PLAGH, Chinese PLA General Hospital; FDUHH, Fudan University Huashan Hospital*.

### *PMP22* Deletions

Twenty-six patients exhibited *PMP22* deletions. These patients shared the typical clinical manifestations, including episodic, recurrent peripheral nerve palsy, which was often preceded by a history of minor trauma at vulnerable sites. The onset age and nerve conduction studies were consistent with the results reported in previous studies. The detection rate was 5.6% in our center and 2.1–2.4% in most CMT centers, except for Huashan Hospital in Shanghai, where the detection rate was 11.5%.

### *PMP22* Point Mutations and Small Deletions

**Figure 3:**
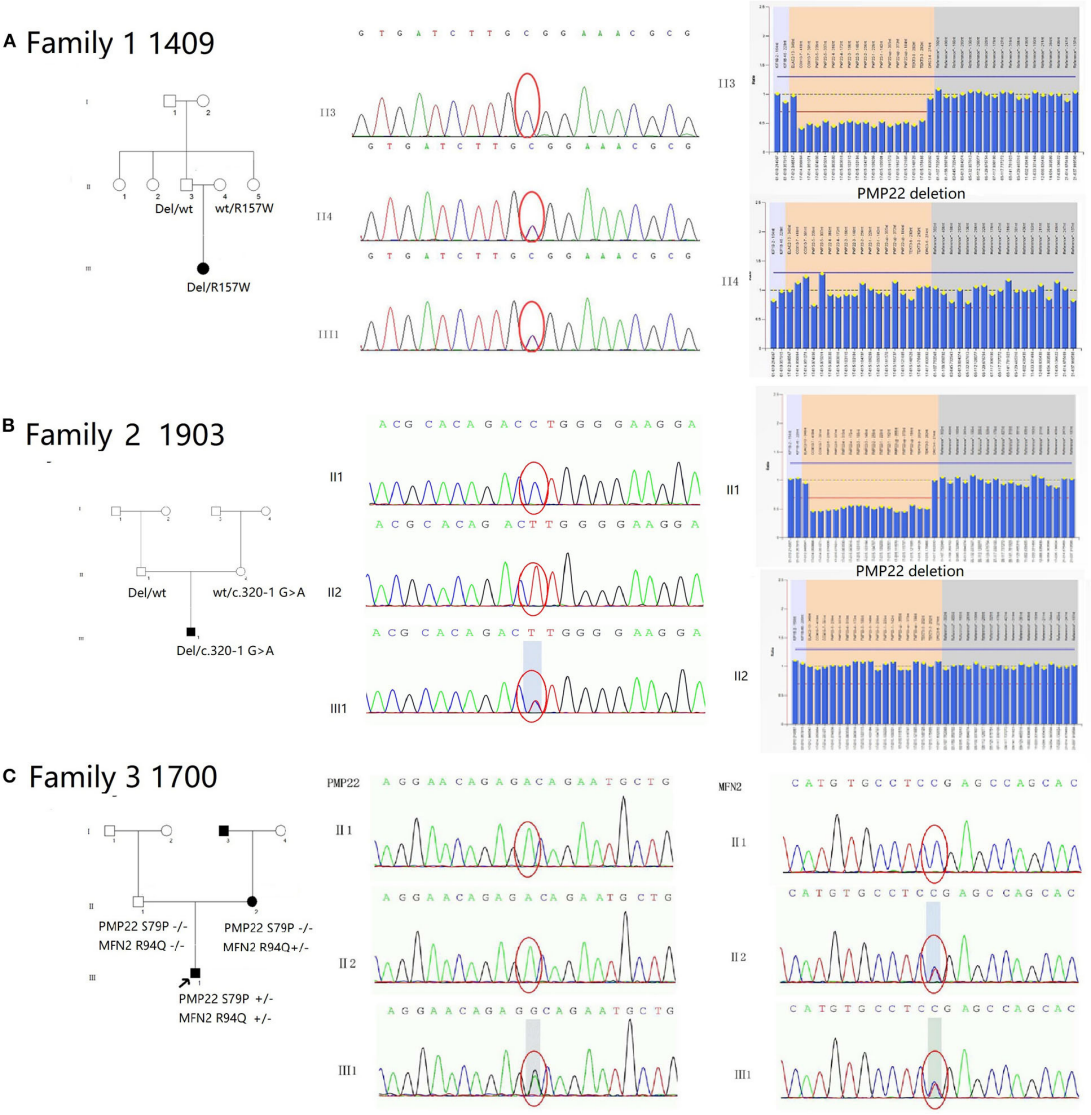
Pedigree and electropherograms of patients with mutations in the *PMP22* gene. **(A)** Family 1 (1409): An affected child was a compound heterozygote for recessive *PMP22* point mutations: the R157W mutation and a 1.5 Mb deletion in 17p11.2-p12. His parents were clinically normal. His father carried a heterozygous deletion of the *PMP22* mutation, and her mother carried the heterozygous mutation R157W. **(B)** Family 2 (1903): An affected child was a compound heterozygote for the recessive *PMP22* novel spicing mutations c.320-1G>A and a 1.5 Mb deletion in 17p11.2-p12. His parents were clinically normal. Her father carried a heterozygous *PMP22* deletion mutation, and her mother carried the heterozygous mutation c.320-1 G>A. **(C)** Family 3 (1700): The heterozygous mutations S79P in *PMP22* and R94Q in *MFN2* were simultaneously observed in proband 1700. The heterozygous R94P mutation in *MFN2* was inherited his mother and the S79P mutation in *PMP22* was a *de novo* mutation.

We observed 10 variants in *PMP22* by screening exons 2, 3, 4, and 5 (Table 3). Two pedigrees presented symptoms of CMT1E (W39C and G107V). The other two pedigrees carried the same previously reported mutation c.434 del T with the classical phenotype of HNPP. Six pedigrees presented symptoms of DSS: two families with recessive mutations carried both *PMP22* deletions and point mutations (the novel mutation c.320-1 G>A and previously reported R157W mutation), which were inherited from their parents separately. Two pedigrees presented the *de novo* mutation of S72W, one of pedigrees presented the *de novo* mutation S79P in *PMP22* concomitantly with the R94W mutation in *MFN2*. We will discuss pedigrees 1490, 1903 and 1700 in greater detail to further analyze the rare genetic mutation (Figure 3).

**Table 3:** Genetic information and clinical features of patients with point mutations in *PMP22*.

**Patient number**	**Mutation**	**Protein change**	**ACMG**	**Phenotype**	**Sex/Age at onset**	**Family history**	**Onset site**	**Skeletal deformities**	**Cranial nerve involvement**	**CMTNS**
1001	c.434 delT	p.Leu145Argfs Ter10	Pathogenic (known)	HNPP	F/38	Mother (+)	Numbness in both hands	(–)	(–)	10
1409	1.5 Mb deletion; c.469C>T	p.Arg157Trp	Pathogenic (known) Pathogenic (known)	DSS	F/7	Father deletion Mother R157W	Weakness in the feet, Pes cavus	Scoliosis	Deafness	27
1523	c.215C>T	p.Ser72Trp	Pathogenic (known)	DSS	M/1	Mother (–) Father(–)	Delayed milestone	(+)	(–)	22
1536	c.320G>T	p.Gly107Val	Pathogenic (known)	CMT1	M/51	Mother (+)	Weakness in the lower extremities	(–)	(–)	13
1537	c.434 delT	p.Leu145Argfs Ter10	Pathogenic (known)	HNPP	M/34	Father (+)	Weakness in the left upper limbs	(–)	(–)	12
1716	c.449G>T	p.Gly150Val	Pathogenic (*de novo*)	DSS	M/at birth	Father (–) Mother (–)	Motor milestone delayed	(–)	(–)	22
1745	c.117G>C	p.Trp39Cys	Pathogenic (known)	CMT1E	F/3	Father (–) Mother (+)	Walking difficulty, foot deformity	(+)	Deafness	26
1700	PMP22 c.235T>C MFN2 c.281G>A	PMP22 p.Ser79Pro MFN2 p.Arg94Gln	Pathogenic (known) (*de novo*)	DSS	M/at birth	Father (–) Mother MFN2 p.Arg94Gln	Motor milestones were delayed	(–)	(–)	22
1903	1.5 Mb deletion; c.320-1G>A	Splicing	Novel	DSS	M/ at birth	Father deletion Mother c.320-1 G>A	Motor milestones were delayed	(+)	(–)	23
1999	c.215C>T	p.Ser72Trp	Pathogenic (known)	DSS	M/1	Mother (–) Father (–)	Delayed milestones	(+)	(–)	20

*Using the family history information of index patients, we confirmed a de novo mutation in 4% (4/10) of PMP22 point mutations*.

#### Pedigree 1409 (Female, 26 Years)

The c.469C>T (R157W) mutation and a deletion in the *PMP22* gene were observed in patient III1 (Figure 3A). The mutations were separately inherited from her parents. The patient suffered from weakness and hypotrophy of her hand muscles at the age of 8 years. After 1 year, she had a clumsy gait and fell easily. These symptoms gradually progressed and became more severe at the age of 14, and she developed hearing problems at this time. When she was examined at 26 years of age, the interosseous hand muscles and distal part of the lower limb were severely affected by pes cavus and hammer toes. The CMTNS score was 27. The electrophysiological studies revealed that motor and sensory nerve conduction velocities were absent in all 4 limbs. Both her parents were healthy and clinically normal; however, the 48-year-old father, who was a carrier of the heterozygous deletion of *PMP22*, had mild bilateral slowing of the motor and sensory conduction velocities and a reduction in the CMAP in the upper and lower limb nerves (ulnar nerve 30.8 m/s, amplitude 3.0 μV; peroneal nerve 46 m/s, amplitude 0.9 μV). Her 46-year-old mother carried a heterozygous c.469C>T mutation. The electrophysiological tests revealed a normal motor nerve velocity but reduced sensory conduction velocity in the median and ulnar nerves (median nerve 17.5 m/s, SNAP amplitude 10.0 μV; ulnar nerve 17.2 m/s, SNAP amplitude 8.3 μV) (Figure 2).

#### Pedigree 1903 (Male, 23 Months)

The splicing mutation c.320-1 G>A and a deletion of the *PMP22* gene were observed in patient III1 (Figure 3B). He presented delayed milestones since the age of 6 months. He was able to turn over at 6 months, sit alone at 12 months and crawl forward at 18 months. He still was unable to walk by the age of 23 months. The mutations were separately inherited from his parents. The neurological examination showed diffuse muscle hypotonia and weakness with absent tendon reflexes. No obvious muscle atrophy was noted. The NCS showed a markedly reduced MCV in the median and ulnar nerves (median nerve 17.1 m/s, CMAP 0.17 mV) and absent response in the peripheral and tibial nerves. No response was recorded in the sensory nerves. Both of his patients were clinically normal. His father carried the deletion mutation in *PMP22* and his mother carried the c.320-1 G>A separately (Figure 2). Their physiological features are compatible with HNPP. The c.320-1 G>A mutation is not present in the EVS, Exac, and dbSNP databases and 650 normal controls, is segregated with the phenotype and is predicted to be most probably affecting splicing by HSF3.0. According to the standard and guidelines of ACMG, it was classified as likely pathogenic.

#### Pedigree 1700 (Male, 18 Months)

The heterozygous mutations S79P in *PMP22* and R94Q in *MFN2* were simultaneously observed in patient 1700 (Figure 3C). His developmental milestones were delayed. He was able to sit alone at 11 months of age, but was unable to stand and walk independently when examined. The neurological evaluation revealed diffuse muscle hypotonia and weakness with absent tendon reflexes. No evidence of muscle atrophies or feet deformities was observed. The electrophysiological studies revealed a lack of the motor and sensory nerve conduction velocities in all 4 limbs. His mother was a patient with CMT2A carrying an R94Q mutation in *MFN2* without the S79P mutation in *PMP22*. She experienced slow progressive weakness, muscle atrophy and sensory loss in the distal limbs since the age of 10 years. When examined at the age of 41, the interosseous hand muscles displayed moderate wasting, the hand muscle strength was reduced to 4/5 (MRC) in the extensors, and the foot flexion and extension were 0/5 and 3/5 (MRC), respectively. A mild diminished sensation in all modalities in the regions below the elbows and knees was observed. His father was clinically and electrophysiologically normal. The molecular genetic testing did not detect any mutations in *PMP22* and *MFN2* (Figure 2).

## Discussion

Five hundred eighty patients from 465 unrelated Chinese families were recruited for this study. We identified 137 index patients with *PMP22* duplications, accounting for 29.5% (137/465) of all CMT cases and 55.7% (137/246) of all demyelinating CMT (CMT1) cases. By comparing the frequency of *PMP22* duplications in our study with the frequencies observed in other CMT centers (Table 2), we estimated that the frequency of *PMP22* duplications in mainland China is ~23.3% (261/1120). The frequency of the *PMP22* duplications was lower than the frequencies reported in Spain (42%) (15), UK (39.5%) (16), the USA (36.5%) (12) and Germany (31.1%) (15, 17); however, the values were similar to those obtained in studies conducted in Japan (15%) (8) and Korea (26.3%) (9) and other CMT centers in China (10, 11). The differences might be attributed to the 3 possible reasons. (1) Ethnic differences or founder effect: Since most Europeans are Caucasians and many American and Australian ancestors are from Northern Europe, the similarity of their results is not surprising. While most Chinese, Japanese and Korean are Mongolians, the low prevalence of PMP22 may result from the founder effect. (2) The clinical features of some Chinese patients may not be sufficiently severe to warrant a detailed examination at hospitals, and hence, the prevalence may be underestimated. (3) Technological limitations may exist; the MLPA has been widely used to detect *PMP22* duplications and deletions, and other methods, including fluorescence *in situ* hybridization and short tandem repeats, which were used in some other CMT centers in China, have been shown to be less sensitive than MLPA. In addition, the difference in the detection rates between the northern and southern parts of China suggest that geographically based differences may also exist, and hence, additional studies with multicenter cooperation are needed.

In our practice, more than half of the patients with *PMP22* duplications presented with symptoms after the age of 20 years. These patients showed a milder disease course than patients with an early onset. These data are consistent with a Japanese study (50%) (8) and a study from Taipei (55.2%) (18), but the values higher than some Western countries (18–26%) (17, 19), suggesting that Mongolian populations may have a mild phenotype due to genetic modifying factors (20) and or environmental factors and may be unaware of being affected. Some patients only had pes cavus, which did not warrant a detailed examination at hospitals, and hence, the prevalence may be underestimated. We also confirmed *de novo* mutations in 3.8% (4/104) of *PMP22* duplications and 40% (4/10) of *PMP22* point mutations, while a study from Germany that included 1330 patients only reported a rate of 1.3% (2/154) for *de novo PMP22* duplications (12). A reasonable assumption is that the low penetrance of inherited neuropathy and highly penetrant *de novo* mutations may also play a role in the lower frequency of *PMP22* duplications.

Deafness in patients with CMT disease is a clinically distinct and rare entity described in several families and is usually associated with a demyelinating neuropathy. Deafness is associated with several point mutations in the *PMP22, MPZ*, and *GJB1* genes (21, 22). Notably, 8.8% (12/137) of index patients with *PMP22* duplications and 20% of index patients (R157W and W39C) with *PMP22* micro mutations had hearing problems. To date, 10 point mutations in the *PMP22* genes result in hearing loss associated with the neuropathy, not including the R157W and W39C mutations identified in our study. The mutations were mostly located in transmembrane domains 1-3 of the protein. The exact pathogenesis of deafness in patients with CMT disease has not been completely elucidated. One recent study revealed that the loss of cochlear Schwann cells may result in permanent auditory deficits characteristic of hidden hearing loss (23). Demyelination of the auditory nerve may be a plausible mechanism to explain the retrocochlear involvement. Auditory thresholds, together with the temporal coding of suprathreshold sounds in speech discrimination and intelligibility, are needed to examine hearing problems in patients with *PMP22* mutations.

We also summarized the largest sample size of patients with *PMP22* point mutations in mainland China. These mutations may result in CMT phenotypes with different severities ranging from DSS to HNPP, depending on the effect of the mutation on the structure and function of the protein (5, 24). Approximately 30 point mutations in *PMP22* have been identified to be related to DSS. Here, we observed 6 index patients who presented symptoms of DSS (G150V, S72W, S79P, R157W, and c.320-1 G>A). G150V has only been identified in a single German study and was submitted to the Clinvar database, but no clinical manifestations and assertion criteria were provided. Our study provides good supporting evidence for the pathogenic effect of this mutation. Two other mutations in the same codon, i.e., G150A (25) and G150D (26), have been reported to cause a severe change in the conserved amino acid residue in the transmembrane domain. Alterations in PMP22 protein trafficking and cytoplasmic retention might be a common mechanism underlying the pathology of dominantly inherited DSS (14, 27, 28), which may cause a dominant-negative effect or a gain-of-function rather than a loss-of-function effect on patients with HNPP.

We have also identified two severely affected patients with DSS who are compound heterozygotes for recessive *PMP22* point mutations (novel mutation c.320-1 G>A and previously reported R157W mutation) and a 1.5 Mb deletion in 17p11.2-p12. Their parents were heterozygous for the *PMP22* point mutations and clinically normal, and presented a only minor impairment in the NCS study, while two others heterozygous for the deletion had HNPP. To our knowledge, only one study has reported the similar compound heterozygous mutation of a T118M point mutation and *PMP22* deletion, suggesting that c.320-1G>A might be another recessive allele contributing to DSS in addition to T118M and R157W (6, 29). The heterozygous mutations c.320 G>T(G107V) and c.320-1G>C in the same codon have previously been reported to be consistent with HNPP (30). Researchers have not clearly determined why T118M, R157W, and c.320-1G>A are severely manifested in a compound heterozygous state in combination with a null allele, since the protein trafficking deficiency was less pronounced in individuals heterozygous for T118M compared to patients with the other heterozygous *PMP22* mutations (S79P and G150C) in the study by Naef and Suter (14). Gain-of-function and dominant negative effects may not be involved. A possible explanation is that those mutations produce a “partial loss-of-function” of *PMP22*. Mice in which both *Pmp22* alleles have been inactivated exhibit severe clinical weakness, markedly reduced nerve conduction velocity, and a severe demyelinating peripheral neuropathy in pathology (12, 31). A study compared the disease severity of individuals heterozygous for T118M with a *PMP22* deletion, homozygous T118M mutation, heterozygous T118M mutation and T118M with *PMP22* duplication, and observed a dose-dependent effect in which a greater amount of remaining PMP22 retained a greater level of function (32).

Patient 1700 carried both the *PMP22* S79P and *MFN2* R94Q mutations. The R94Q mutation in *MFN2* was dominantly transmitted from his mother, and the S79P mutation in *PMP22* was a *de novo* mutation. R94Q is considered a hotspot in the *MFN2* gene because its substitution has been described independently in more than 30 studies, thus representing the most frequently mutated residue in *MFN2*. Mutations in arginine 94 usually cause severe CMT2 with an onset between 2 and 7 years of age. Additional symptoms, particularly optic atrophy, a late onset, and a milder phenotype of axonal neuropathy, have also been observed. Since his mother had a relatively late onset (10 years) and a much milder phenotype (she could walk and use a pen at the age of 41 years), the severe clinical symptoms in patient 1700 may be attributed to the S79P mutation in *PMP22*. However, we cannot exclude the effect of the *MFN2* mutation in combination with *PMP22*. To the best of our knowledge, this report is the first to describe 2 severe disease-causing mutations in *PMP22* and *MFN2* in a single patient. Due to the rapid progress in NGS, WES, and WGS sequencing techniques, an increasing number of mutations have been identified in different genes, thereby the determination of an accurate diagnosis is more complicated. Additional studies are needed to determine whether the two severe disease-causing mutations exert separate, modified or combined effects on the protein. Therefore, the clinical phenotype should be considered in combination with the genetic results, emphasizing the importance of a careful examination of the medical history to determine an accurate diagnosis.

## Conclusions

We conducted a study to provide the most detailed description of *PMP22*-related neuropathy in mainland China. The phenotypic variability highlights the importance of considering *PMP22* mutations in a broad spectrum of demyelinating neuropathies. The c.320-1G>A mutation might be another recessive allele contributing to severe forms of CMT1 in addition to T118M and R157W. The results provide valuable insights into the current strategy of genetic testing for CMT disease. NGS technology has increased the potential for the efficient detection of variants of unknown significance (VUS) and concurrent causative genes. Greater cooperation between neurologists and molecular biologists is needed in future investigations.

## Data Availability Statement

The datasets generated for this study can be found in the http://www.mono-mybg.com/jzjy-cmt.

## Ethics Statement

The studies involving human participants were reviewed and approved by The Institutional Ethics Committee of Peking University Third Hospital (PUTH) approved this study (IRB 00006761). Written informed consent to participate in this study was provided by the participants' legal guardian/next of kin. Written informed consent was obtained from the individual(s), and minor(s)' legal guardian/next of kin, for the publication of any potentially identifiable images or data included in this article.

## Author Contributions

DF and XL conceived and designed the study. XD provided valuable clinical materials. YZ performed the genetic testing. XL wrote the paper. DF reviewed and edited the manuscript. All authors read and approved the manuscript.

## Conflict of Interest

The authors declare that the research was conducted in the absence of any commercial or financial relationships that could be construed as a potential conflict of interest.

## Abbreviations

AD: Autosomal dominant
AR: autosomal recessive
ACMG: American College of Medical Genetics
CMAP: compound muscle action potential
CMT: Charcot-Marie-Tooth
CMTES: Charcot–Marie–Tooth examination score
CMTNS: Charcot–Marie–Tooth neuropathy score
DSS: Dejerine-Sottas neuropathies
DM: diabetes mellitus
EMG: electromyography
GDAP1: ganglioside-induced differentiation-associated protein 1
GJB1: gap junction protein beta-1
HNPP: hereditary neuropathy with liability to pressure palsies
MFN2: mitofusin2
MLPA: multiplex ligation-dependent probe amplification
MPZ: myelin protein zero
NGS: next-generation sequencing
PMP22: peripheral myelin protein 22
SNAP: sensory nerve action potential
VUS: variants of unknown significance.
